# Effects of heme precursors on CYP1A2 and POR expression in the baculovirus/Spodoptera frugiperda system^☆^

**DOI:** 10.1016/S1674-8301(10)60034-6

**Published:** 2010-05

**Authors:** Huiyuan Lu, Jun Ma, Nian Liu, Shoulin Wang

**Affiliations:** Key Lab of Modern Toxicology of Ministry of Education, School of Public Health, Nanjing Medical University, Nanjing 210029, Jiangsu Province, China

**Keywords:** CYP1A2, POR, baculovirus/*Spodoptera frugiperda* system, co-expression, heme precursors

## Abstract

**Objective:**

CYP1A2 and NADPH-CYP450 oxidoreductase (POR) were expressed in the baculovirus/*Spodoptera frugiperda (sf9)* system. The aim of this study was to investigate the effects of heme precursors on the expression of CYP1A2 and POR.

**Methods:**

The heme precursors [δ-Aminolaevulinic Acid (5-ALA), Fe^3+^ and hemin] were introduced into the system to evaluate their effects on the expression of CYP1A2, POR and their co-expression. All the proteins were identified using immunoblotting, CO-difference spectroscopy, or cytochrome c assay.

**Results:**

In the present study, functional CYP1A2 and POR were successfully expressed in the baculovirus/*sf9* system, and both of them showed high activities. Co-addition of 5-ALA and Fe^3+^ significantly improved expression of CYP1A2 by about 50% compared with the addition of 5-ALA, Fe^3+^ or hemin alone. Either co-addition of 5-ALA and Fe^3+^ or addition of 5-ALA or Fe^3+^ alone improved the POR expression level 2 fold and its activity 7-10 fold compared with control (no addition). However, unlike CYP1A2, there was no difference between the co-addition and addition of these heme precursors alone. Different ratios of BvCYP1A2 to BvPOR also affected the co-expression of CYP1A2 and POR, with a 3:1 ratio of BvCYP1A2 / BvPOR significantly increasing their co-expression. Surprisingly, the addition of 0.1 mM 5-ALA or Fe^3+^ alone, but not their co-addition, could significantly improve the CYP1A2 and POR co-expression (*P* < 0.05).

**Conclusion:**

5-ALA and Fe^3+^ increased the expression of CYP1A2 and POR in a baculovirus/*sf9* system, but the pattern of their expression was different between their expression alone and co-expression.

## INTRODUCTION

Cytochrome P450 (CYP450) monooxgenases, make up 70%-80% of all phase I xenobiotic-metabolizing enzymes. They can metabolize a large number of endogenous compounds and are involve in many cellular functions[1]. The CYP450s superfamily consists of many subfamilies, including CYP1A, 2A, 3A, 1B, 1C, and so on. CYP1A2 belongs to the CYP1A subfamily, which constitutes approximately 13% of the total liver CYP content[2]. CYP1A2 is involved in the metabolism of several endogenous compounds and widely used drugs, and in the activation of procarcinogens such as aflatoxin B1, the commonly recognized hepatocarcinogen[3]–[4]. Since CYP450s are a group of biocatalysts, sources of electrons are necessary for their activation. The addition of two electrons (reduction) to the heme iron of CYP450s may break the hard oxygen-oxygen bond. NADPH-CYP450 oxidoreductase (POR) is a single protein which transfers electrons from NADPH to CYP450s in the endoplasmic reticulum[5]–[6]. It is usually regarded as the partner of the CYP450s in the metabolism of xenobiotics and endogenous compounds.

Although the primary structures of CYP450 enzymes are well known, studies on functional properties of different eukaryotic CYP450s have lagged behind because of the lack of efficient systems for heterologous expression of catalytically active enzymes[7]. A number of systems, including both prokaryotic (*e.g.*, *E.coli*) and eukaryotic (*e.g.*, yeast, insect cells, and mammalian cells) systems have been used to express mammalian CYP450s. Among these, the Bac-to-Bac baculovirus expression system usually yields the highest level of expression of CYP450s because the baculovirus shuttle vector in this system has an integrity genome of *Autographa californica* multiple nuclear polyhedrosis virus (AcMNPV, Ac) and can increase the expression efficiency through site-specific transposition[8]. In addition, *Spodoptera frugiperda (sf9)* insect cells are very important for the efficient expression of CYP450 because they have similar modes for post-transcription and post-translation of proteins to mammalian cells. As a result, the baculovirus/*sf9* expression system can yield high levels of unmodified, native CYP450 proteins, except for the noted deficiency of *sf9* cells in heme incorporation[8]. Thus the addition of a heme precursor is necessary to compensate the baculovirus/*sf9* system in order to maximize the expression of the functional CYP450s.

CYP450s is a heme-containing protein, so the heme iron in CYP450s plays a very important role in their catalytic activations. Heme refers to the ferrous iron (Fe^2+^) bound to tetrapyrrole, a macrocyclic porphyrin, and its oxidized form (Fe^3+^) is known as hemin. Hemin can provide heme to synthesize CYP proteins in the baculovirus/*sf9* system. δ-Aminolaevulinic Acid (5-ALA) is the first committed intermediate of the heme biosynthesis pathway *in vivo*, and ferric citrate can provide ferrous iron for this pathway as well as heme for the baculovirus/*sf9* system. POR should be taken into consideration for P450s heterologous expression because POR content is rate-limiting for CYP reactions[9]. Previous studies showed that functional CYP450s could be obtained by adding cofactors (*e.g.*, 5-ALA[10], ferric citrate, or hemin[11],[12]), and the approach of POR and CYP450s co-expression via co-infection enabled the production of enzymatically active CYP450s in the natural microsomal membrane[10]. These methods provide possible ways to reconstitute catalytically active systems *in vitro*. Because of the complication of CYP450s expression *in vitro*, systematic studies should be undertaken to find the most favorable conditions for stable and efficient expression of CYP450s.

In the current study, the baculovirus/*sf9* system was utilized for the expression of CYP450s, POR, and their co-expression. The influences of different cofactors such as hemin, 5-ALA and Fe^3+^ were considered to optimize the expression system. This would help to provide a reliable research model for constructing the metabolism of xenobiotics *in vitro*.

## MATERIALS AND METHODS

### Reagents

Ferric citrate, 5-ALA, cytochrome *c*, and a monoclonal anti-CYP1A2 antibody were purchased from Sigma-Aldrich (St. Louis, MO, USA). *Sf9* insect cells, Bac-to-Bac baculovirus expression system, Cellfectin® reagent, unsupplemented Grace Insect Cell Culture Medium and SF900 II SFM were obtained from Invitrogen (Carlsbad, CA, USA). Rabbit anti-human POR polyclonal antibody was from Millipore (Bedford, MA, USA). Anti-mouse IgG, horseradish peroxidase-linked whole antibody (from sheep), and the enhanced chemiluminescence (ECL) western blot detection reagents were purchased from Cell Signaling Technology (Danvers, MA, USA). Emulgen 911 was obtained from Karlan Research Products Corporation (Cottonwood, AZ, USA). Solid sodium dithionite was purchased from Sinopharm (Shanghai, China).

### Expression of human CYP1A2 and POR proteins in the baculovirus/Sf9 system

All the proteins were expressed in the Bac-to-Bac baculovirus expression system (Invitrogen, Carlsbad, CA, USA) following the manufacturer's instructions. Briefly, the CYP1A2 and POR cDNAs were first cloned into a pFastBac™ 1 vector to construct the donor vectors, and then were transformed into MAX Efficiency® DH10Bac™ chemically competent cells (Invitrogen, Carlsbad, CA, USA) to form the recombinant bacmid which were subsequently used to transfect the *sf9* cells to produce recombinant baculovirus particles, and further to get the baculoviral stock via infecting the *sf9* cells. Ferric citrate (0.1 M in double distilled water) and/or 5-ALA (0.1 M in SF900 II SFM medium), or hemin (2 mg/ml in 50% ethanol and 0.2 M NaOH) was then added to the culture medium. After 72h-incubation, the infected *sf9* cells were harvested, washed twice with 0.1 M potassium phosphate buffer (pH 7.4) and re-suspended in the reaction buffer [0.1 M potassium phosphate containing 20% (V/V) glycerol, 1 mM fresh PMSF, 0.1 mM EDTA, 0.1 mM DTT, 0.5%(V/V) Emulgen 911]. The microsomes were prepared by sonicating with an Ultrasonic Processer (CPX130, Cole-Parmer, USA) and centrifuging as described elsewhere[13]. The prepared microsomes were stored at -80°C before use.

### P450 content determination

The P450 content was determined by reduced CO-differential spectra following the previous study[14]. Microsomal proteins were diluted to 1 mg/ml with the reaction buffer. The protein solution was placed in a 1 cm optical path cuvette and reduced by a few milligrams of solid sodium dithionite. After the base-line was recorded, the sample cuvette was bubbled with CO for 60 sec. The difference in spectrum absorption was measured by scanning from 550 nm to 400 nm using an UV/visible spectrophotometer (DU® 800, Beckman coulter, USA).

### POR activity determination

Cytochrome c assay was used to determine the activity of POR. In brief, 840 µl reductase assay buffer (containing 0.05 M potassium phosphate, 0.1 mM EDTA, 0.3 M KCl, pH7.4) was put into a 1cm optical path cuvette, and then about 20 µl microsomal protein (dilute about 30 times) and 100 µl 0.8mM cytochrome *c* were added. After the base-line was recorded, 50 µl 4 mM NADPH was added and mixed immediately to start the reaction. The spectrophotometric rate was measured at 550 nm.

### Immunoblot analysis

Immunoblotting for measuring CYP1A2 and POR protein levels was carried out as described previously[15]. Microsomal proteins were separated by SDS-polyacrylamide gel electrophoresis, transferred to a nitrocellulose sheet, and then incubated with a monoclonal antibody against human CYP1A2 as the primary antibody (1:5000), followed by the binding of a secondary antibody (goat anti-mouse IgG, 1:2000) conjugated with horseradish peroxidase. In order to detect POR, a polyclonal antibody against human POR was used as the primary antibody (1:2000) and an anti-rabbit IgG conjugated with horseradish peroxidase as the secondary antibody (1:2000). The immunoblots were visualized using ECL detection according to the manufacturer's protocol. For the densitometric analysis, the protein bands on the blot were measured using BandScan 4.3 software.

### Statistical analysis

Data are expressed as mean±SD. Statistical differences were analyzed using one-way analysis of variance (ANOVA), and the LSD method was used for multiple comparisons. P-values less than 0.05 were considered statistically significant.

## RESULTS

### Construction of recombinant pFastBac™ 1-CYPs (POR) and Ac-Bacmid-CYPs (POR)

**Fig. 1:**
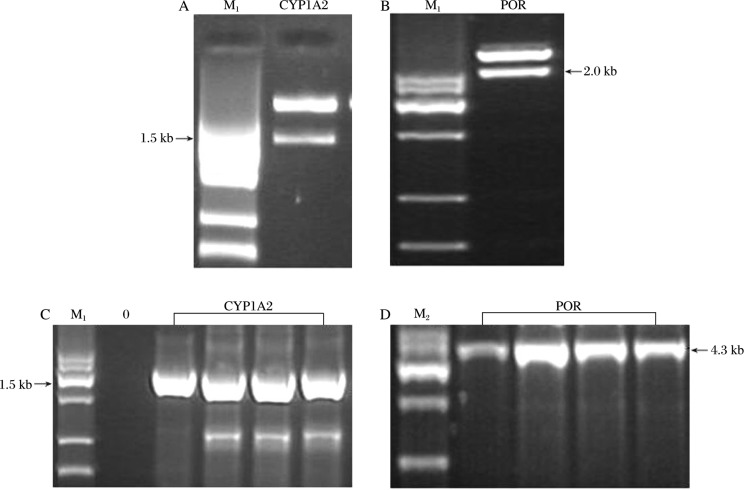
Identification of the recombinant transposing plamid by restriction endonulease digestion and PCR. M_1_: Marker VII. M_2_: Marker IV. A and C: *CYP1A2* gene. B and D: *POR* gene.

Recombinant plasmids of pFastBac™ 1-CYPs (POR) were obtained via double digestion by restriction enzymes and Quik T4 DNA ligase and verified by restriction endonuclease analysis. The recombinant plasmid pFastBac™ 1-CYP1A2 digested with Kpn I/XbaI generated a 1.5 kb fragment (total length of *CYP1A2* gene is about 1.5 kb, GeneBank No.: NM_000761) and a 4.7 kb fragment (equal to the length of vector, ***Fig.1A***), while pFastBac™ 1-POR digested with BamH I/Kpn I generated a 2.0 kb fragment (total length of *POR* gene is about 2.0 kb, GeneBank No.: NM_000941) and a 4.7 kb fragment (***Fig.1B***). Reconstructed Ac-Bacmid-CYPs (POR) were obtained by transforming pFastBac™ 1-CYPs(POR) into the bacmid of competent DH10Bac cells and verified by PCR. The results showed that Ac-Bacmid-CYP1A2 generated a 1.5 kb fragment (using primers of 1A2 Forward: 5′-ATGGCATTGTCCCAGTCT GTTCC-3′ and 1A2 Reverse: 5′- TCAATTGATGGA-GAAGCGCCGCGCCTG-3′, ***Fig.1C***), and Ac-Bacmid-POR generated a 4.3 kb fragment (using primers of M13 Forward: 5′-GTTTTCCCAGTCACGAC-3′ and M13 Reverse: 5′-CAGGAAACAGCTATGAC-3′, ***Fig.1D***). Finally, the reconstructed Ac-Bacmid-CYPs (POR) were transfected into *sf9* cells to obtain recombinant baculovirus particles.

### Heterologous expression of human CYP1A2 and POR in baculovirus/*sf9* system

**Fig. 2:**
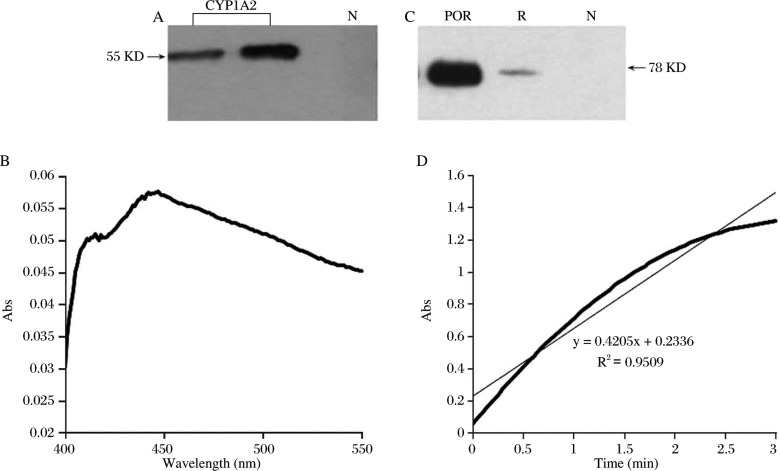
Heterologous expression of recombinant CYP1A2 and POR cDNA in *sf9* cells. Immunoblots of CYP1A2 (A) and POR (C). B: CO difference spectroscopy analysis of CYP1A2. D: cytochrome *c* assay of POR. N: negative. R: prepared rat liver microsomes.

*Sf9* cells were infected by CYP1A2 or POR recombinant baculovirus particles, and then the traditional conditions were used to express these proteins: ferric citrate and 5-ALA stock solutions were added to cell culture medium with a final concentration of 0.1 mM. Infected cells were harvested after an incubation time of 72h. Immunoblot analysis detected a single protein band with the expected molecular weight (55KD and 78KD respectively) (***Fig.2A and 2C***). In the CO-difference spectroscopy analysis, CYP1A2 protein displayed a characteristic absorption peak at 450 nm (***Fig.2B***) and the content was 0.21 nmol/mg protein. Cytochrome *c* assay revealed that POR activity was 1,519.47 unit/mg protein.

### Effects of different factors on CYP450s (POR) expression

**Fig. 3:**
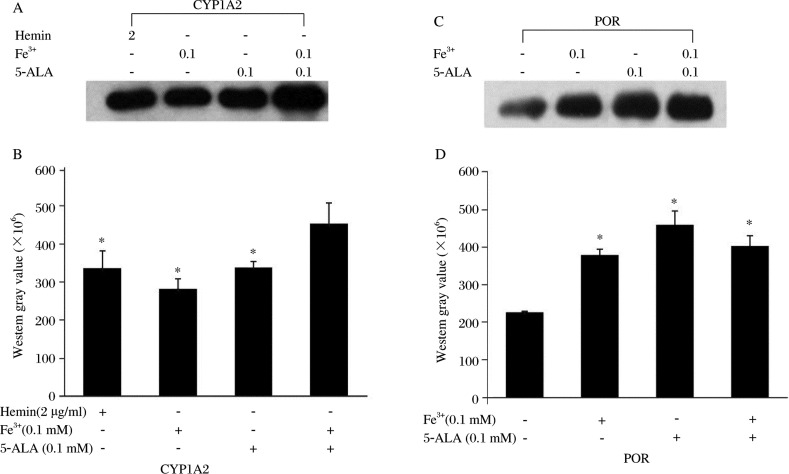
Effects of different factors on CYP1A2 and POR expression. A: CYP1A2. B: POR Microsomal proteins (5 µg) of each sample were used. The results are expressed as western gray scale values (mean±SD; *n* = 3). *P < 0.05, compared with the corresponding groups (B), or compared with the group without adding factors (D).

To establish the optimal conditions of the heme precursors (hemin, 5-ALA and Fe^3+^) for expression, CYP1A2 and POR expression were measured in various combination of 0.1 mM 5-ALA, 0.1 mM Fe^3+^ or 2 µg/ml hemin. Our results showed that compared with adding 5-ALA, Fe^3+^ or hemin alone, co-adding 5-ALA and Fe^3+^ could improve CYP1A2 expression significantly (35%, 51% and 35% higher, respectively) (***Fig. 3A and 3B***). However, no significant difference was found when adding 5-ALA, Fe^3+^ or hemin alone. The POR expression level was about double control (adding nothing) when 5-ALA and/or Fe^3+^ were added (***Fig. 3C and 3D***). Similarly, the POR specific activities in 5-ALA, Fe^3+^ alone or together were 7.1, 4.5, 5.4 times higher than adding no factors (***Table 1***). Compared with the addition of 5-ALA or Fe^3+^ alone, the addition of both together did not increase the expression level and activity of POR (*P* > 0.05), which differed from CYP1A2.

**Table 1:** Effects of different factors on POR activity

Factors (0.1mM)	Microsomal protein conc. (mg/ml)	ΔA_550nm_ / min	Activity (Unit)	Specific Activity (Unit/mg microsomal prep.)
0 (control)	8.099	0.037	1.99	122.81
5-ALA	10.31	0.380	20.51	994.62
Fe^3+^	7.54	0.190	10.28	681.42
5-ALA and Fe^3+^	10.89	0.316	17.07	783.50

### Effects of different virus ratios on co-expression of CYP1A2 and POR

**Fig. 4:**
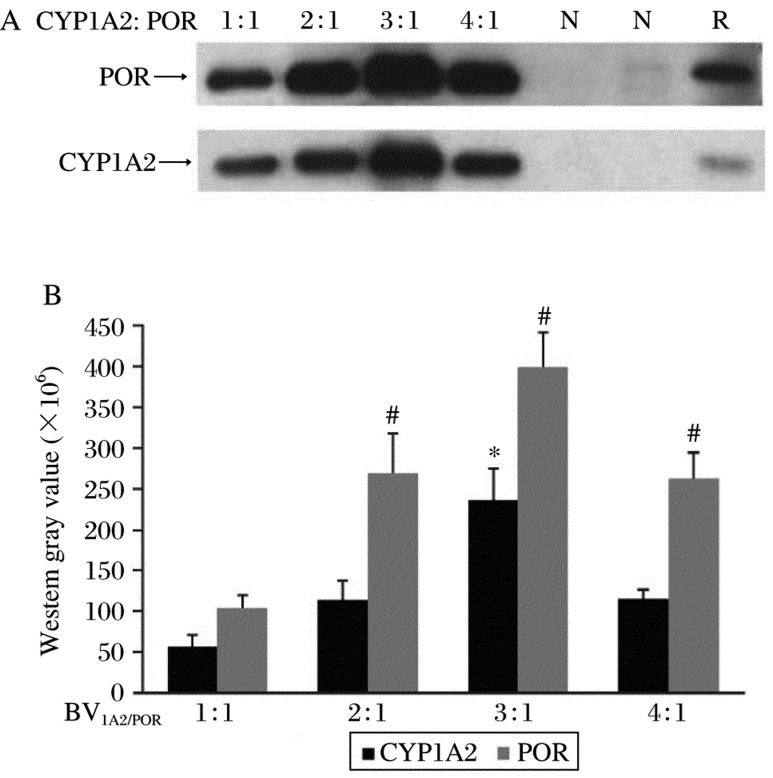
Co-expression of CYP1A2 and POR using different virus ratios. Microsomal proteins (5 µg) of each sample were used. The western blot results (A) were expressed by western gray scale values (B) (mean±SD; *n* = 3), which defined the CYP1A2:POR virus ratio of 1:1 as the control, *P < 0.05 of CYP1A2, ^#^*P* < 0.05 of POR.

CYP450s and POR could be co-expressed in *sf9* cells by co-infecting with two recombinant viruses, bvCYP450s and bvPOR. This method had the advantage that it controlled the CYP/POR ratio by changing the ratio of bvCYP450s to bvPOR[10]. To obtain an optimal ratio of CYP/POR recombinant virus, we tried different ratios of bvCYP/bvPOR to investigate their effects on co-expression. Immunoblot analysis revealed that the bvCYP/bvPOR ratio affected the co-expression greatly. It showed that CYP1A2 and POR expression levels were significantly higher when the CYP1A2:POR virus ratio was 3:1 compared to other ratios (***Fig. 4A and 4B***).

### Effects of different factors on co-expression of CYP1A2 and POR

**Fig. 5:**
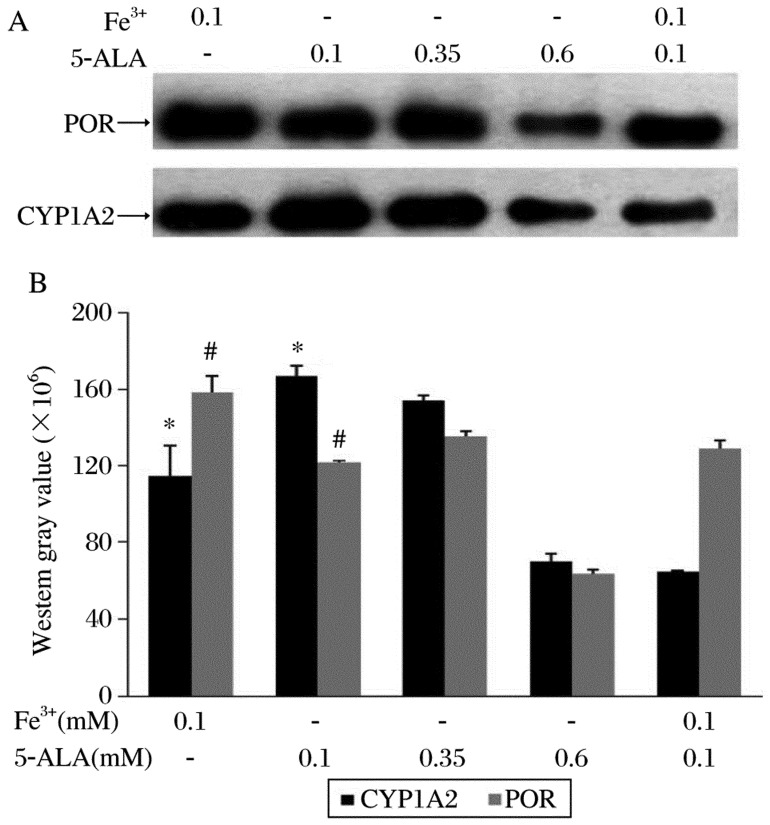
Effects of different factors on co-expression of CYP1A2 and POR. Microsomal proteins (5 µg) of each sample were used. The western blot results (A) were expressed by western gray scale values (B) (mean±SD; *n* = 3). *P < 0.05 of CYP1A2, ^#^*P* < 0.05 of POR, both compared with the group treated with 0.1 mM Fe^3+^ and 0.1 mM 5-ALA.

To optimize the conditions for co-expression of CYP1A2 and POR, the CYP1A2 : POR virus ratio (3:1) was selected to detect the effects of various concentrations of 5-ALA (0.1-0.6 mM) and/or ferric citrate (0.1 mM) on the co-expression. Immunoblot analysis showed that low concentrations of 5-ALA (0.1 mM, 0.35 mM) favored the co-expression of CYP1A2 and POR. 5-ALA at both concentrations resulted in higher expression than did 0.6 mM 5-ALA. Unlike the effect of 5-ALA and Fe^3+^ co-addition on the CYP1A2 or POR expression alone, this co-addition could not increase the CYP1A2 and POR co-expression level which was significantly lower than that induced by adding 5-ALA or Fe^3+^ alone (*P* < 0.05). (***Fig. 5A and 5B***).

## DISCUSSION

Because of the importance of CYP450s in metabolizing endogenous and exogenous compounds, attempts have been made to express them in heterologous systems, including mammalian cells, yeasts and bacteria[16],[17]. However, current expression systems for producing eukaryotic P450s are hampered by serious drawbacks such as ① the low efficiency of production; ② the requirement of expensive materials and facilities, as well as sophisticated methods; ③ the extensive degradation of enzyme by the expression host or even the absence of biological activity. The baculovirus/*sf9* system has been considered most efficient expression system. But, *sf9* cells have a noted deficiency in CYP450s expression, namely their incorporation of heme. This problem can be partially resolved by adding heme precursors into the culture medium[18],[19].

Hemin is the oxidized form (Fe^3+^) of heme. It can increase heme biosynthetic enzyme activity and stimulate other enzymes in the heme biosynthetic pathway. Hemin was used to express functional CYP1A1[18], CYP2A13[11], CYP2E1[12], CYP2S1[20] in baculovirus expression systems. However, hemin may be toxic to *sf9* cells that have been in culture for several months[8]. 5-ALA is the first compound in the porphyrin synthesis pathway that leads to heme in mammals and to chlorophyll in plants, and the last step of this pathway is the combination of protoporphyrin IX with iron. It has received attention for its great potential as a holodiagnostic agent in clinical practice, and as a selective and biodegradable herbicide, insecticide and growth promoting factor[21]. It also has medical applications in photodynamic cancer therapy and tumor diagnosis[22]. Additionally, 5-ALA is the first committed intermediate of the heme biosynthesis pathway, which has been used in the medium of *E.coli* to express CYP450 1A1[23] and its allelic variants[24], CYP21, CYP1A2[7], as well as co-expression of CYP1A1 variants and POR[10] in *sf9* cells.

In our study, CYP450s in *sf9* cells were expressed successfully after adding hemin, 5-ALA and (or) Fe^3+^. The highest expression level of CYP1A2 in experiments using the combined addition of 5-ALA and Fe^3+^ revealed that the *sf9* cells had cell substructure integrity and all the enzyme pathways necessary for heme synthesis. Our results also revealed that as the addition of Fe^3+^ improved the expression, it might be that iron is essential for the synthesis pathway. For this reason the cell medium was supplemented with the addition of Fe^3+^ as an iron source. Also the level of CYP1A2 with the co-addition of 5-ALA and Fe^3+^ was higher than that with hemin alone. The reason might be the toxicity of hemin to *sf9* cells. Moreover, the method used to dissolve the hemin (dissolved in 0.2 M NaOH and 50% ethanol) might affect the enzyme expression because NaOH and ethanol could change the pH value of the culture medium, which subsequently decreased the availability of *sf9* cells and then the protein expression. Although heme was not essential for POR expression and activity, these were significantly higher with 5-ALA or (and) Fe^3+^ than without any factors. As we mentioned above, 5-ALA is a growth promoting factor and it may promote the growth of *sf9* cells. Thus it can increase both POR and CYP1A2 expression levels and activities.

The electron transfer chain, usually NADPH and POR, plays an important role in reconstituting catalytically active CYP450 systems for metabolic activation. Therefore co-expression of CYP450 and POR in the baculovirus/*sf9* system was studied extensively because this system could enable high production of the unmodified, enzymatically active native CYP450 in the microsomal membrane. Previous studies revealed successful expression of CYP1A1, CYP2A1 and CYP2A6, but their co-expression levels with POR were 50%-60% lower than their expression alone[10],[25],[26]. As discussed in these papers, it was assumed that POR expression, probably via heme degradation, directly influenced the CYP450 holoenzyme level. One study on co-expression of CYP2D6 and POR using an expression vector containing both CYP2D6 and POR cDNAs revealed that high levels of POR having an adverse effect on CYP450 expression might be a general property of co-expression in sf9 cells[27],[28]. During the co-expression, degradation of the expressed holo-CYP1A1[10], CYP2E1 and CYP3A1[29] could be circumvented, at least partially, by addition of heme ligand or inhibitors such as imidazole and α-naphthoflavone, because these inhibitors can strongly bind to microsomal CYP by a direct ligation of an azole nitrogen with the iron atom of the CYP heme group[30]. The current study provided a new way to increase co-expression levels of CYP and POR by changing the CYP/POR ratio and combination of heme precursors. However, the co-expression level decreased with the simultaneous addition of 5-ALA and Fe3+, which did not affect the expression of CYP1A2 or POR alone. The mechanism of this phenomenon should be further explored.
